# Beyond the Textbook: A Reflective Journey Through Humanity and Community Based Medical Learning

**DOI:** 10.31729/jnma.v64i293.9294

**Published:** 2026-01-31

**Authors:** Shubham Shrestha, Aaditya Rimal

**Affiliations:** 1 Patan Academy of Health Sciences, Lagankhel, Lalitpur, Nepal

**Keywords:** *CBLE*, *communication skills*, *experiential learning*, *medical humanities*, *reflection*

## Abstract

Medical education extends beyond textbooks, with experiential learning crucial to cultivate empathy, professionalism and social accountability. Our curriculum at the Patan Academy of Health Sciences includes simulations of being differently abled, medical humanities, communication skill development and community and rural clinical postings. Compassionate communication developed along with critical thinking and growth as a professional when interacting with elderly residents, chronic diseases patients and Female Community Health Volunteers. These reflections highlight the transformational impact of experiential learning in shaping full and competent physicians.

## INTRODUCTION

The real life care of a patient often presents challenges that textbooks cannot capture. At the Patan Academy of Health Sciences (PAHS), the curriculum has specifically been designed to incorporate experiential learning from the outset. Students engage themselves in structured activities such as simulations of being differently abled, reflective literature exercises, communication skill training and community and rural postings.

These experiences linked theoretical knowledge to practice and exposed students to social, cultural and environmental realities that impinged on health outcomes. These promote empathy and critical thinking skills that underpin good, locally accountable and responsive medicine on an individual and population level which go well beyond biomedical expertise alone.^1,2^ These experiences from our medical college are reflected in this article.

## HUMANITY AND EMPATHY IN MEDICAL LEARNING

At PAHS, a different approach is taken at the start of medical school. Instead of jumping into the basic sciences, a eight week long foundational course is begun which includes many different disciplines geared towards welcoming students into the world of medicine and patient care. One of those subjects is medical humanities. Putting ourselves in the shoes of the differently abled, via navigating the busy streets near our medical college and visiting a busy local tea shop, the lack of accessibility was laid bare to us; forcing us to think about how these challenges affect healthcare access and overall quality of life. The exercise creates empathy, patience and much more of an understanding of the barriers patients face.^3^

It is recognized that empathy is crucial for the physician patient relationship and aids in positive health outcomes.^4^ Reading reflective literature like When Breath Becomes Air; furthered introspection on mortality, professional values and patient centered care.

Death and the concept of a ‘good death’ was often discussed during our medical humanities education. Via poems, plays and the creation of short videos we explored these themes to explore what they meant to us, not just physically, but also spiritually. Even on a personal level, such exercises really made us grateful for our own lives and the great venture we were on to preserve that for others. This is also backed in the data as a significant rise in empathy scores have been seen after the medical humanities course that is based around the first two years of medical school at PAHS.^5^

## COMMUNITY AND RURAL CLINICAL EXPERIENCES

Among the most transformative parts of our training was the Community Based Learning and Education (CBLE) and the rural postings. It was here that we witnessed the complex interaction of social, cultural and environmental issues that make up a person’s health. When asked about hand washing practices during the COVID pandemic an elderly grandmother remarked that despite the Female Community Health Volunteer’s (FCHV’s) requests for more frequent hand washing, the fact that they barely had enough water to drink made it impossible to spare water for the act.

Her words exposed the gap between the medical textbook recommendations and the patient’s real life, emphasizing social, environmental and economic constraints on whether health advice can realistically be followed.

Later as we moved to more rural postings, we lived with local people to learn about their barriers to health. It was common to live hours away from the nearest health post. We visited a family with unvaccinated children; the four years old son had profound developmental delays and had been seen by only a faith healer. There are few accessible early interventions for such developmental conditions, which when available, are extensive and expensive. This brought home for us that the gulf between sophisticated medical care in urban and rural areas was immense. What about this poor boy, who missed out on the early intervention he needed? How will his life look in a place where his condition will be blamed on spirits and bad omens rather than a neurobiological view which might help him live a healthy life?

In the rural hospitals postings we saw that doctors served as caregivers, counselors and advocates. A woman with a mild fever requested antibiotics, citing an aunt that got better with the drugs. By explaining that viral illnesses wouldn’t benefit by medications, we were able to avoid overtreatment, establish trust, and reassure her. This demonstrated the general responsibility of physicians to manage anxiety, ensure that patients are well led while maintaining a caring but rational practice approach.

These immersive experiences taught us the value of contextual understanding, empathy and culturally sensitive communication. Living and working in resource limited settings, where networks and infrastructure may not be present, made us realize daily hardships both patients and communities go through, shaping our way of approaching practical and patient centered care.

## REFLECTIONS ON TRANSFORMATIVE EXPERIENCES

These lessons have shown that no textbook could prepare students entirely for the demands of patient care. As we near the end of medical school, we encounter patients lives in a more practical way. In Out Patient Departments (OPD), we don’t just look at the symptoms but remember their living conditions and means. Respect is something we owe for their presence, and it humbles us as future doctors. Agitated patients, long waits, overfull wards all serve as the reminders to ourselves about empathy and de-escalation. Our experiences teach us to promote patients relief from suffering rather than just cure of disease and invite patient centered care. This model helps maintain the trust between individuals and hospitals and the health system grows sustainably and inclusively to serve all of Nepal’s communities.

## WAY FORWARD

Medical curricula should integrate experiential learning into traditional biomedical education. Going beyond the textbook and empathizing with patients with respect to their challenges and where they come from should be mandatory to learn. Other ways to enhance professional development include reflective exercises, mentorship and longitudinal community engagement.

Such a curriculum would ensure that graduates are clinically competent, empathetic and socially accountable in the management of complex healthcare realities. This enables physicians to cure with care, heal when cure is impossible and treat patients as holistic individuals rather than objects of sympathy.

## References

[1] Frenk J, Chen L, Bhutta ZA, Cohen J, Crisp N, Evans T (2010). Health Professionals for a New Century: Transforming Education to Strengthen Health Systems in an Interdependent World.. The Lancet..

[2] Wear D, Zarconi J (2008). Can Compassion Be Taught? Let’s Ask Our Students.. J Gen Intern Med..

[3] Branch WT (2015). Teaching Professional and Humanistic Values: Suggestion for a Practical and Theoretical Model.. Patient Educ Couns..

[4] Decety J (2020). Empathy in Medicine: What It Is, and How Much We Really Need It.. Am J Med..

[5] Arjyal A, Gc KB, Subedi M, Gongal RN (2024). A Longitudinal Study on Change in Empathy in the First Two Years among Medical Students.. J Patan Acad Health Sci..

